# A Case of Intus-Susception of the Colon, with Remarks

**Published:** 1864-03

**Authors:** W. Gould

**Affiliations:** Buffalo


ART. IV.—*A Case of Intus-Susception of the Colon, with Remarks.* BY W. GOULD, M. D.

A. B., aged three months, twenty-four days, born in Buffalo, American parents. On Sunday, January 10, 1864, his mother while washing and dressing him noticed a peculiar deathly sickness, which lasted but a short time, and then passed off. Nothing was thought of this until the following Wednesday, when it commenced passing blood from the bowels, and vomited its food as soon as taken. On Thursday, 14th, I was called to see it. It was passing blood freely at short intervals, and vomited whenever it took the breast. Was restless, looked bright, but the countenance indicated much distress, Its bowels had been regular from its birth up to this attack on Wednesday, but during the twenty-four hours preceding my visit, it had passed nothing but blood. I was unable to make a satisfactory diagnosis.

Treatment consisted of acetate plumbi, hydrarg. chlor. mite a a, grs. j, sach. qs. M ft. pulv. vj. S. One powder every hour until the vomiting ceased. And as the discharge from the bowels was attended with considerable pain and effort, two drops tr. opii in a small quantity, of starch water was ordered as an enema as often as the discharge occurred. Tinct. opii comp. was also directed to be given, to relieve the pain and restlessness, in doses sufficient for that purpose.

Friday, 15th. The discharge of blood continues. The vomiting has ceased. Bowels slightly tympanitic. With these exceptions the symptoms and appearances are about the same. As the vomiting had ceased and the bowels had not moved, gave hyd. ch. mite and rhei. a a, grs. ii, to be repeated every two hours until three doses were taken, and then followed with injections of ol. racini and soap suds. This proved unavailing, no fecal discharge followed. My little patient was evidently sinking. Although the discharge of blood was less, the bowels were more tympanitic, and there was evidently some obstruction of the bowels, but the nature of that obstruction I was certainly at a loss to determine. It might be something swallowed, or impacted feces. The symptoms and progress of the disease in so young a child, were new to me; yet the result was more and more evident to end in death.

Sunday, 17th. My little patient died at 8 A. M. I asked and obtained permission to make a post mortem examination, which was done on Monday, 18th, twenty-seven hours after death, Dr. Gay assisting me. On opening the abdomen, with the exception of a little congestion upon the external surface of the descending colon, the appearance was healthy. In the rectum could be felt a hard mass, which was supposed to be fecal matter, but upon placing a ligature below it, and raising the intestines it slid back some eight or ten inches to its normal position, and proved to be a mass of strangulated intestines, which had been forced down within two inches of the anus. Immediately above the invagination we found stricture of the colon to such a degree as to preclude all possibility of relief from any treatment beyond the palliative. The accident occurred above the sigmoid flexure, and near the angle formed by the transverse and descending colon, In a subject so young the bands of muscular fibre which produce the peculiar saculated charactar of the colon is not so marked as in that of adults. To me this case is instructive and interesting,

First—On account of its inevitable fatality.

Second—On its occurrence in one so young.

Third—The peculiarity of its location, in the colon.

Fourth—That no similar case is to be found on record, so far as I can learn, in this city.

Fifth—Cases elsewhere, and what authors say upon the subject.

Upon these several points I propose to make a few remarks, and should I digress a little from the exact order, as noticed above, I trust it will be pardoned.

1st.—In regard to its fatality. It must rank as one of the worst and most dangerous affections to which infants or even adults are liable. Could we possibly in the case of a child only three or four months old, be enabled to make a correct diagnosis, would any treatment beyond the palliative be of use? The answer will be in the negative, and the reason will appear obvious to any one who examines the specimen preserved by me, and notices the large mass of the invaginated portion and the narrow stricture immediately above.

2d,—Its occurrence in one so young. I was induced to examine with much circumspection the reports of the several Health Physicians of this City, and certainly expected to find many cases of adults, if not of children. I was surprised to find but one case reported from June, 1855, to May, 1859, and of course unable to ascertain the age of the victim, or whether the cause of death was determined by a *post mortem* or, as I fear is too frequently the case, guessed at. Here the question occurs to me as to how many of the deaths reported under the head of "*Ignotus*," ranging yearly from five to forty, may have died from this accident. Of course the question cannot now be answered satisfactorily, and I will frankly say that only for the privilège of making a post mortem examination, so kindly granted by its parents, I would now be in a degree ignorant of the true cause of my little patient's death.

3d.—The peculiarity of its occurring in the colon. Authors say that it may take place in any part of the alimentary canal, while all agree that it most generally occurs in the ilium, near where it terminates in the colon, caused by augmented peristaltic or spasmodic action, forcing a portion of the intestines into the lower or conversely. The first termed "progressive," and the latter "retrogade," which is rare, undoubtedly. As an evidence that it often occurs in the death struggle, it is affirmed that in 300 cases of children who died in a French hospital from worms, or fever, the greater part had from two to four or more intus-susceptions. In these cases it is said that the symptoms were absent during life, and in all probability were caused by the death struggle, and only found in the small intestines.

4th.—That no similar case is upon record, so far as I can ascertain, in this city. I regret that more accurate reports have not been made in former years by our Health Physicians. I find that during the first ten years Dr. Flint published the Buffalo *Medical Journal* no reports appear. In April, 1855, the Health Physician, Dr. John Root, commenced and is entitled to the credit of first making monthly reports to that Journal. They were continued until 1860, when the *Journal* became a New York City institution and the reports ceased. In July, 1861, the Buffalo *Medical and Surgical Journal* made its appearance, and for a time we had the "reports," but we look in vain for them now, and here let me ask, why is this? Are the monthly reports of city mortality of so little account to the profession that a journal published in our midst for the ostensible purpose of information should be denied these reports and your subscribers have to cut them from the daily papers and keep the scraps for future reference instead of having them in the *Journal* where they certainly belong? One of two conclusions I think inevitable. Either the present Health Physician is not aware that we have a *Medical Journal* in Buffalo, or he is indifferent as to whether the profession have his reports in a paper they preserve or not. Another reason may be that the Editor is indifferent as to giving them a place. Perhaps it costs a little too much time and labor, both so precious, to obtain them. Whatever the cause of omission may be, I trust it will be removed and we shall have the reports in the *Journal* in future.

5th.—Cases elsewhere and what authors say upon the subject. I see that in Philadelphia from 1834 to 1840 there are 1881 deaths reported of children under one year. Nine are reported as having died from invagination of the intestines. Whether this fact was determined from symptoms or *post mortem* examinations does not appear. This ratio would give one in just 209 from this cause. If this is true in Philadelphia why not in Buffalo. The term "*Ignotus*" may include many from this cause. Coley, in his work on "Diseases of Infants and Children," barely mentions its occurrence as a *sequel* of Dysentery.

Condie speaks of it as "often met with in children who have died of other diseases and appears to take place in the act of dying, from some convulsive or inordinate movement of the muscular fibres of the intestine canal. These investigations give rise to no symptoms during the life-time of the patient; and after death are reducible with perfect ease. Occasionally, however, the invagination occurring in children gives rise to symptoms of the most serious character, and speedily destroys the life of the patient." He also says that the invagination may take place in any part of the canal, but is most generally seated near the termination of the ilium and that the disease is generally fatal, in fact that all cases falling under his observation, terminated fatally. That so far as treatment is concerned, palliatives are only of use. Where the case is strongly marked and the symptoms are unmistakable the operation of Saparotomy has been by some recommended with the view of restoring or disentangling the invaginated portion of the bowels, but the practice should be condemned for obvious reasons. Still another point of interest in this case is to determine at what time the accident or act of invagination occurred. My own theory is that it happened as far back as Sunday, 10th, and in all probability at the time his mother noticed his apparent sickness while being washed and dressed. The absence of stercoraceous vomiting is accounted for when we remember the locality of the lesion so near the extremity of the canal. I believe when this occurs early in the disease it is an evidence that the invagination is seated in the small intestines, and *vice versa.*

**Buffalo, February 22d, 1864.**

